# Gradual emergence of spontaneous correlated brain activity during fading of general anesthesia in rats: Evidences from fMRI and local field potentials

**DOI:** 10.1016/j.neuroimage.2015.03.037

**Published:** 2015-07-01

**Authors:** Ruggero G. Bettinardi, Núria Tort-Colet, Marcel Ruiz-Mejias, Maria V. Sanchez-Vives, Gustavo Deco

**Affiliations:** a Center for Brain and Cognition, Computational Neuroscience Group, Universitat Pompeu Fabra, Barcelona 08018, Spain; b Institut D' Investigacions Biomèdiques August Pi i Sunyer (IDIBAPS), Barcelona 08036, Spain; c Institució Catalana de Recerca i Estudis Avançats (ICREA), Barcelona 08010, Spain

**Keywords:** BLCs, band-limited correlations, RCNs, robust coupled nodes, Brain states, Connectivity, Anesthesia levels, Default-mode network, Alpha rhythm, Gamma rhythm

## Abstract

Intrinsic brain activity is characterized by the presence of highly structured networks of correlated fluctuations between different regions of the brain. Such networks encompass different functions, whose properties are known to be modulated by the ongoing global brain state and are altered in several neurobiological disorders. In the present study, we induced a deep state of anesthesia in rats by means of a ketamine/medetomidine peritoneal injection, and analyzed the time course of the correlation between the brain activity in different areas while anesthesia spontaneously decreased over time. We compared results separately obtained from fMRI and local field potentials (LFPs) under the same anesthesia protocol, finding that while most profound phases of anesthesia can be described by overall sparse connectivity, stereotypical activity and poor functional integration, during lighter states different frequency-specific functional networks emerge, endowing the gradual restoration of structured large-scale activity seen during rest. Noteworthy, our *in vivo* results show that those areas belonging to the same functional network (the default-mode) exhibited sustained correlated oscillations around 10 Hz throughout the protocol, suggesting the presence of a specific functional backbone that is preserved even during deeper phases of anesthesia. Finally, the overall pattern of results obtained from both imaging and *in vivo*-recordings suggests that the progressive emergence from deep anesthesia is reflected by a corresponding gradual increase of organized correlated oscillations across the cortex.

## Highlights

• Rat brain activity was recorded while anesthesia spontaneously decreased over time.
• Fading of anesthesia modulates the overall pattern of brain structured co-activations.
• Areas of the same network show frequency-specific coupling even in deep anesthesia.
• Light anesthesia is characterized by the gradual emergence of large-scale connectivity.
• Correlated band-limited oscillations distinguish between states and network properties.

## Introduction

The intrinsic complexity of brain organization allows the emergence of a wide range of different activity regimes, referred to as brain states. Ongoing brain activity during waking rest exhibits spontaneous dynamics that are characterized by highly structured patterns of correlated fluctuations known as Resting State Networks (RSN, Biswal et al., 1995; Greicius et al., 2003; Fox et al., 2005; Beckmann et al., 2005; Deco et al., 2011; Cabral et al., 2014a). In recent years, a growing number of studies have indicated the differences in spontaneous dynamics underlying different brain states, as during sleep (Horovitz et al., 2008; Larson-Prior et al., 2009), anesthesia (Kaisti et al., 2002; Boveroux et al., 2010), meditation (Brewer et al., 2011; Hasenkamp and Barsalou, 2012; Tang et al., 2012), psychedelic states (Vollenweider and Kometer, 2010; Carhart-Harris et al., 2012, 2013), and also at different states of brain development (Fransson et al., 2007, 2009). Ongoing activity observed during anesthesia and light sedation (Greicius, 2008; Stamatakis et al., 2010) shows intriguing similarities with slow-wave sleep (Horovitz et al., 2009). Moreover, it has been proposed that many mechanisms underlying anesthesia-induced loss of consciousness are also implicated in the fading of consciousness characterizing the descent to sleep (Franks, 2008; Brown et al., 2010). Many authors have investigated RSN in animals under general anesthesia (Lu et al., 2007; Pawela et al., 2008; Hutchison et al., 2010; Liu et al., 2011; Tu et al., 2011) and during wakefulness (Liang et al., 2011; Zhang et al., 2010), revealing the existence of intrinsic brain networks in primates (Mantini et al., 2011; Dawson et al., 2013) and rodents (Becerra et al., 2011; Lu et al., 2012). The results obtained so far suggest that deeper stages of anesthesia tend to be characterized by diminished functional connectivity (Lu et al., 2007; Williams et al., 2010; Wang et al., 2011), and that the nature of such decrease is related to the anesthetic agent used (Pawela et al., 2008; Liu et al., 2013). Nonetheless, the modulation of large-scale connectivity during the spontaneous fading from a deep state of anesthesia to a lighter one is still unclear. Investigations of brain states have largely relied on the region-specific metabolic demands related to neural activity, which is at the basis of imaging techniques such as positron-emission tomography (PET, Raichle, 1980) and functional magnetic resonance imaging (fMRI, Ogawa et al., 1992; Ogawa and Sung, 2007), characterized by high spatial accuracy but limited temporal resolution. The application of high temporal resolution techniques such as electroencephalography (EEG), magnetoencephalography (MEG), and electrocorticography (ECoG) and intracortical recordings such as local field potentials (LFPs) has been crucial to elucidate the finer temporal structure of brain activity, revealing that different global states are linked to specific rhythms in humans and animals (Steriade et al., 1996; Buzsáki and Draguhn, 2004; Buzsáki, 2006). A significant portion of brain structural architecture is phylogenetically conserved in vertebrates (Striedter, 2005), with fundamental similarities among mammals (Hofman, 1989). This inter-species similarity in anatomical connectivity gives rise to the emergence of comparable patterns of organized activity, usually referred to as functional networks (for a review see Park and Friston, 2013). The primary objective of this paper is to investigate how different brain states consistently modulate network functionality in the rat, both at the macroscopic (fMRI) and mesoscopic (LFPs) scales, and by means of comparing the connectivity between areas pertaining to the same or different network. Our results confirmed that different states of anesthesia are mirrored by broad changes in the underlying functional organization that occur at different spatio-temporal levels, and that the state-related emergence of large-scale functional networks is sustained by inter-areal correlated oscillations at specific frequencies. Additionally, our findings suggest the existence of a frequency-specific association between correlated activity as measured with fMRI and LFPs.

## Materials and methods

### Animal preparation

Animals were deeply anesthetized by intraperitoneal injection of ketamine (60 mg/kg) and medetomidine (0.5 mg/kg). Brain activity was recorded from the deepest phase of anesthesia up to partial recovery. Descent and full recovery were not recorded, as they are experimentally demanding stages that often lead to artifacts. We excluded the possibility of recording full emergence from anesthesia also because the animal was not chronically implanted. The animals were continuously monitored by controlling the respiratory pattern in imaging and the heart rate during *in vivo* experiments. The animals were not paralyzed and the hind paw reflexes were regularly tested during electrophysiological recordings (see below). Atropine (0.05 mg/kg) was injected subcutaneously to prevent secretions. Body temperature was maintained at 37 °C using a water-circulating heating pump (T/Pump, Gaymar, USA). Animal age, sex, weight and body fat are factors known to modify the anesthesia metabolism, thus animals were selected that exhibited similar characteristics (all adult Wistar males, 293 ± 43 g). All the procedures were carried out in compliance with the European Community Council Directive for the care and use of laboratory animals (86/609/ECC) and with the Generalitat de Catalunya's authorization (DOGC 2450 7/8/1997, Comite Ético de Experimentación Animal, Universidad de Barcelona).

### fMRI recordings

MRI experiments were conducted on a 7.0 T BioSpec 70/30 horizontal animal scanner (BrukerBioSpin, Ettlingen, Germany), equipped with a 12 cm inner diameter actively shielded gradient system (400 mT/m). The receiver coil was a phased-array surface coil for the rat brain. Each anesthetized animal (*n* = 5) was placed in the prone position in a Plexiglas holder with a nose cone for administering a mixture of 30% O2 and 70% N2, and were fixed using a tooth bar, ear bars and adhesive tape. The animals were not paralyzed during the procedure. Tripilot scans were used to ensure the accurate positioning of the animal's head in the isocenter of the magnet. Echo planar imaging (EPI) sequence started 40 min after anesthesia induction and was continuously acquired over a period of around 2.3 h with the following conditions: echo time (TE) = 50 ms, repetition time (TR) = 3 s, field of view (FOV) = 25.6 × 25.6 × 7 mm, matrix size = 64 × 64 × 7 pixels, resulting in a spatial resolution of 0.4 × 0.4 × 1 mm. Additionally, for recording purposes a T2 weighted anatomical image was acquired by using a RARE (Rapid Acquisition with Refocusing Echoes) sequence and the following parameters: TE = 11 ms, TR = 1.6 s, and same FOV, matrix size and spatial resolution as above. We obtained 7 coronal slices 2 mm thick. The resulting images were then treated in order to obtain the maximum number of isolated brain areas. Consequently, a given number of regions of interest (ROIs) were then obtained from each rat. Images were not treated for motion correction, as they presented stable positioning and alignment along the entire experiment. The selection of ROIs and corresponding spatial normalization was performed by comparing MRI images with a rat-brain atlas (Paxinos and Watson, 2004), taking into account the following criteria: first, the selected areas had to contain at least four voxels per image, but in no case could those in the limit of the area contain borders of brain or cortex or confounding limits between areas; secondly, the area had to be present in at least 80% of the voxels. The reference slices were the ones presenting medial prefrontal cortex area in the rostral side, the one presenting primary visual cortex (V1) in the caudal side, and one central slice where the hippocampal structures were identified. The intermediate slices were treated taking these previous three as a reference and identifying structures such as hippocampal formation, ventricles and corpus callosum as well as different subcortical structures. These two criteria limited the number of ROIs, which in every animal was the maximum number of regions that satisfied these objectives. Those criteria allowed the extraction of BOLD (Blood Oxygen Level Dependent) signal from 14 ROIs from each hemisphere, leading to a total of 28 ROIs in each of the 5 animals. The extracted ROIs were the primary motor cortex (M1), primary and secondary somatosensory cortices (S1, S2), primary and secondary visual cortices (V1, V2M), primary auditory cortex (A1), medial prefrontal cortex (mPF), retrosplenial cortex (Rspl), cingulate cortex (CC), thalamus (Thal), striatum (Str), amygdala (Amy), hippocampus (Hipp) and hypothalamus (Hyp). ROIs and average BOLD signals were extracted with homemade scripts implemented in Matlab (Mathworks, Natick, MA, USA). In order to discard physiological ultra-slow fluctuations of the BOLD signal (Yan et al., 2009), while maintaining those that had been previously shown to be relevant for sampling low-frequency rat brain functional networks (Hutchison et al., 2010), we removed the best-fitting linear trend from the BOLD traces and band-passed them at 0.01–0.1 Hz. Obtained signals were then standardized.

### *In vivo* LFP recordings

**Fig. 1:**
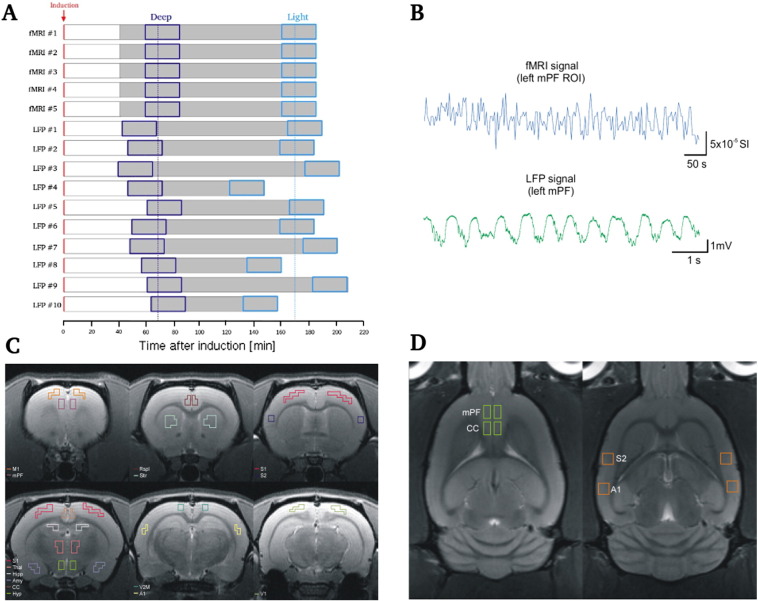
Experimental protocol and recorded areas in imaging and *in vivo* experiments. (A) Schematic representation of the experimental protocol used in fMRI and LFPs experiments (see Materials and methods). The red arrow indicates the moment of anesthesia induction, whereas the recorded intervals are highlighted in gray. Dark and light blue boxes indicate the intervals used as representative of deep and light anesthesia, respectively (see Identification of intervals corresponding to deeper and lighter states of anesthesia). Overall (fMRI and LFPs), deep intervals were centered at 65.4 ± 9.2 (mean ± SD) minutes, whereas light intervals were centered at 171 ± 17.7 min after induction, as indicated by the dark and light blue dotted lines. (B) Examples of BOLD (Blood Oxygen Level-Dependent, blue) and extracellular local field potential (LFP, green) of the activity in medial prefrontal cortex (mPF). (C) Regions of interest (ROIs) used for BOLD signal extraction in imaging experiments. (D) Regions that were selected for LFPs. MPF = medial prefrontal cortex; CC = cingulate cortex; S2 = secondary somatosensory cortex; A1 = primary auditory cortex.

Lidocaine was administered at all pressure points and incisions prior to surgery. Approximately 30 min after induction, while the anesthesia was deepest, craniotomies were performed to record from the left medial prefrontal cortex (mPF, 3.2 mm AP, 0.8 mm ML) and left and right cingulate cortex (CC, + 1 mm AP, + 0.8 mm ML) in 10 rats, and to record from the left primary auditory cortex (A1, − 5.2 mm AP, + 6.5 mm ML) and the left and right secondary somatosensory cortex (S2, − 1.3 mm AP, + 5.6 mm ML) in 6 animals. All coordinates are relative to bregma (following Paxinos and Watson, 2004; see Fig. 1D). Extracellular slow-wave recordings were obtained with tungsten electrodes of impedances of 1–2 MΩ (as in Ruiz-Mejias et al., 2011). Electrodes were placed in infragranular layers (3 mm deep in mPF, 2.4 mm in CC, 2.4 mm in A1 and 3.4 mm in S2). Recordings were amplified with a multichannel system (Multichannel Systems, Germany) and the signal was digitized and acquired at 10 KHz with a CED acquisition board and Spike2 software (Cambridge Electronic Design, UK). Local field potentials of the selected cortical areas were simultaneously recorded in the anesthetized rat, using the same anesthesia protocol as in imaging experiments. Extracellular recordings started 49 ± 9 (mean ± SD) minutes after induction (depending on the time needed for surgery) and continued until the animal exhibited weak withdrawal reflex after hind paw pinch. Recordings before and after hind paw stimulation were discarded to avoid artifacts, resulting in a total duration of LFPs recordings of 135 ± 26 (mean ± SD) minutes. Obtained signals were down-sampled offline to 333 Hz, and a notch filter was then applied in order to remove 50 Hz electrical noise. At the end of the experiment the animals were administered a lethal dose of sodium pentobarbital (800 mg/kg).

### fMRI data analysis

Preprocessed BOLD signal was divided in 90% overlapping sliding windows of 10 min (corresponding to 200 sample points each), each one shifting one minute in time with respect to the previous one. The use of sliding windows to evaluate time-varying changes in brain activity and connectivity have been successfully reported in some recent works, both on humans and on anesthetized animals (Hutchison et al., 2013; Keilholz et al., 2013; Thompson et al., 2013). BOLD signal variability over time of each individual ROI was evaluated computing the variance of the preprocessed time-series in each sliding window, whereupon the mean BOLD variability was calculated as the mean variance of each sliding window across all areas. The results were then averaged across rats. According to Friston et al. (1993), functional connectivity (FC) was calculated using pairwise Pearson's correlation between each pair of areas of each sliding window. Since the sampling distribution of the Pearson's correlation coefficients is known to be non-normal, the values were converted to their corresponding *Fisher's z-scores* in order to be properly compared (Fisher, 1915). The mean correlation time course was then computed averaging all the 378 traces ((28 × (28 − 1))/2) obtained from all area pairs. Similarly, we calculated the distribution of the correlation coefficients (and its standard deviation) in each sliding window. We then computed, for each sliding window, the mean Kuramoto order parameter χ, a measure that describes the degree of overall network synchronization in a system of coupled oscillators (Kuramoto, 1975, see *Supplemental Methods*). This measure quantifies the uniformity of the phases across all the nodes in the network at a given time, ranging from 0 for a fully incoherent network state and 1 for a fully synchronized one. In order to promote efficient information processing, the outputs of different and highly specialized computations have to be coordinated and integrated into distributed but coherent neural activity. In the present work, we were interested in evaluating if the ability of the brain to integrate information across distant regions changed during the gradual fading of anesthetic effect. We can define functional integration as the transient binding of information across brain regions, a phenomenon that can be measured from the overall structure of correlations between areas in a given temporal window. To this aim we applied, on the correlation matrix obtained from each sliding window, a measure of functional integration based on the size of the largest connected component (see description below), thus obtaining a picture of the time-varying changes in functional integration taking place over the course of anesthesia. First, we binarized the Pearson's correlation matrix obtained from each 10-minute sliding window by applying a threshold *T* ranging from 0 to 1 with subsequent steps of 0.01 and following the criterion that if |*r_ij_*| < *Τ* we set 0, and 1 otherwise. Thus for each sliding window we obtained as many binarized adjacency matrices *A_ij_*(*T*) as the number of applied thresholds. We then calculated the size of the largest component of each *A_ij_*(*T*). In this framework, a network component is defined as a sub-network in which all edges are connected to each other by paths, but are not connected to any additional vertex of the supergraph. The size of a network component is given by the total number of its vertices. The size of the (largest) component is thus a measure related to the (upper bound in the) amount of correlated activity that is integrated within a set of connected vertices. In order to get a measure independent of the threshold, we then integrated that curve within the range of the threshold between 0 and 1.

### Identification of intervals corresponding to deeper and lighter states of anesthesia

Imaging measurements robustly indicated the presence of a continuous and non-monotonic progression over time from a stage characterized by low BOLD variability and weakly correlated activity to one marked by higher BOLD variability and stronger and more heterogeneously correlated fluctuations. According to this pattern, we were able to take as representative of a deeper phase of anesthesia the 15 sliding windows starting 60 min after induction, whereas the last 15 windows were selected as representative of light anesthesia. The sliding windows used to calculate the changes of a given measure over time all had same length (10 min), which means that the intervals used to statistically compare the two phases of anesthesia were 25 min each, *i.e.* long enough to sample substantial and reliable differences between brain states. In contrast to fMRI, during LFPs experiments the level of anesthesia could be more precisely assessed by checking the absence of the withdrawal reflex tested with hind paw pinch (see In vivo LFP recordings). On average, LFPs deep intervals were centered at 61.8 ± 10 (mean ± SD) minutes after induction, whereas the light ones spanned a larger range, 171.7 ± 22 min (see Fig. 1A). Henceforth, we will refer to the above mentioned intervals as deep and light anesthesia, respectively.

### Identification of robust coupled nodes and uncoupled area pairs

We used the BOLD signal corresponding to the two anesthesia intervals to compute stationary functional connectivity matrices and the corresponding correlation distributions. Results were then Fisher transformed (*see* Results). For further analysis we focused only on the correlations between those pairs of areas that simultaneously satisfied two constraints in all five rats: First, *r_ij_* had to be significantly different from zero (with *p* < 0.05); secondly, such correlation had to have comparable values across rats, *i.e.*, the standard deviation (SD) of its average across all rats had to be smaller than 1, which means a maximum standard error (SE) of 0.4, being the usual SE value for biological phenomena below this threshold. We referred to the obtain subset of area pairs as Robust Coupled Nodes (RCNs). Area pairs that didn't show correlation significantly different from zero either in deep and light intervals were considered “uncoupled”.

### Detection of functional communities and modularity

We computed the community structure of RCN sparse matrices obtained from light anesthesia applying a version of Louvain's community-detection algorithm based on a modularity function for weighted networks (Blondel et al., 2008; Gómez et al., 2009), implemented in the Brain Connectivity Toolbox (Rubinov and Sporns, 2010). Modularity Q is a cost function (see *Supplemental Methods*) used to optimize community detection. It evaluates the “goodness” of a partition by counting the total number of edges falling within groups compared to the expected number of edges that may fall within the groups in equivalent networks whereby edges are placed at random (thus preserving the total number of edges in each node). Modularity optimization leads to graphs partitioned into densely intraconnected groups of nodes (referred to as “communities”), which are sparsely connected between them. Modularity can thus be considered a measure of segregation. Louvain's community detection method is stochastic, and its results can vary from run to run. For this reason we ran the method 10,000 times for each sparse RCNs network, and then defined the most probable community to which each area pair belonged as the community to which it was most often assigned over the 10,000 repetitions. The community structure computed on individual rats' light RCN matrices always returned the same community partition, indicating inter-subject consistency and confirming the reliability of the selected RCNs. We thus isolated two areas (mPF and CC) that, according to our analysis, belonged to same functional community, and considered them as an example of “coupled” regions, *i.e.* brain areas whose activities are reliably connected as they take part to the same functional network. In order to capture the overall changes in communities' structure taking place during the gradual fading of anesthesia from the individual to the group level, we first iterated 10,000 times the community detection algorithm on the full (28 × 28) FC matrix obtained from each sliding window of each animal, and then computed the *a posteriori* probability that each pair of ROIs had of belonging to the same community across all iterations and all animals in each sliding window. We thus calculated, for each one of the obtained probability matrices, the mean *a posteriori* probability of belonging to the same community, in order to obtain a rather simple summary of the overall changes in the structural stability of different communities over the course of anesthesia. After that, we measured functional segregation over time computing the modularity index Q from the FC matrix obtained from each sliding window, and then average results across animals.

### LFPs data analysis

In order to evaluate the frequency-specific coupling between coupled (mPF-CC) and uncoupled (A1-S2) regions in deep and light anesthesia, we computed the correlation between the envelopes of homologous band-limited signals obtained from two different regions, to which we refer to as band-limited correlations (BLCs). BLCs can be seen as an extension of the classical functional connectivity (Friston et al., 1993) in the frequency domain and can be used to quantify the degree of co-variation between neuronal oscillations of two distant cortical regions at a given frequency (Brookes et al., 2011; Hipp et al., 2012; Cabral et al., 2014b). To this aim, the continuous down-sampled data was bandpassed in 100 non-overlapping frequency bands (1 Hz width) from 0 to 100 Hz. After having calculated the Hilbert envelopes of the 100 signals obtained for each area and having applied a squared-log transformation in order to ensure normality of the estimates (as done in Hipp et al., 2012), we band-passed the resulting envelopes at the same resolution used in our previous imaging experiments (0.01–0.1 Hz) and down-sampled them at 1 s to account for the low-frequency components of the oscillations classically sampled in fMRI experiments. Then, for each animal we computed the Pearson's correlation coefficient between the resulting signals from each homologous frequency band between pairs of areas and converted it into the corresponding Fisher's *z-score*, resulting in 100 correlation values for coupled and uncoupled areas (one *per* frequency band). We then computed the difference between the BLCs of the two states (deep and light) for each 1 Hz-width frequency band for coupled and uncoupled area pairs (see *Supplemental Methods*). Subsequently, we divided the original preprocessed LFP signal in 90% overlapping windows of 10 min, as previously done for fMRI data. We then calculated BLCs in each sliding window. BLC time courses of the broader classical frequency bands were calculated analogously after having band-passed the preprocessed LFP signal according to the following frequency ranges: slow waves (0.01–1 Hz), low δ (1–2 Hz), high δ (2–4 Hz), θ (4–8 Hz), α (8–15 Hz), β (15–30 Hz), low γ (30–50 Hz) and high γ (50–100 Hz).

### Quantification of the frequency shift to higher frequencies

During BLC analysis we observed the presence of two shifts to higher frequencies in correlated oscillations during the gradual transition from deep to light anesthesia, the first shift around 1 Hz and the second around 10 Hz (see Results). To investigate this aspect, we computed the relative correlation in the two frequency bands of interest (~ 1 Hz and ~ 10 Hz) as the ratio between the higher and the lower component of a given frequency-band-of-interest over sliding windows for a given area pair (see *Supplemental Method*). This procedure quantifies how much the mean band-limited correlation in a given frequency range varies in relation to another frequency range, thus making it possible to measure their relative contributions in different intervals of time. Values greater than 1 indicate predominance of the higher frequency BLC component, whereas values smaller than one indicate the opposite (1 indicates perfect balance). For each animal, we calculated the relative correlation in deep and light intervals for the transition from < 1 Hz to 1–2 Hz and that from 8–10 Hz to 11–15 Hz in both coupled (*n* = 10) and uncoupled (*n* = 6) areas, and then average across rats.

## Results

### Correlation between BOLD time courses increases as anesthesia gradually fades

**Fig. 2:**
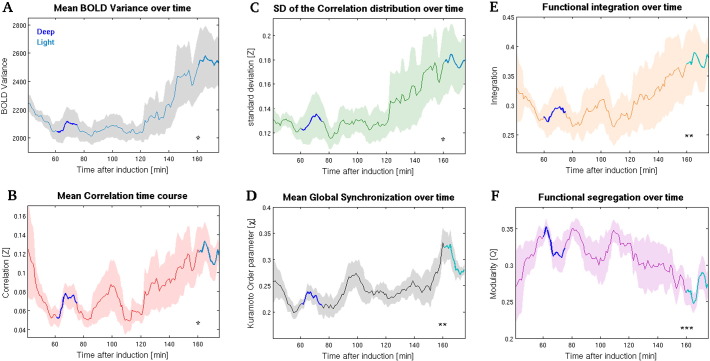
The progressive fading of anesthesia is associated with gradual changes in overall brain activity. (A) Time course of the mean BOLD variance across all areas confirms that decreasing levels of anesthesia are mirrored by increased BOLD variability. (B) Mean correlation over time computed over all area pairs. (C) Standard deviation (SD) of the correlation distribution over time. (D) Mean global synchronization (Kuramoto order parameter, χ) over time. Not surprisingly, this metric resembles the mean correlation across all areas, suggesting that the level of anesthesia modulates the overall network's ability to synchronize. (E) Functional integration over time. (F) Change of modularity (Q) over time. Modularity is a measure of functional segregation and, as expected, follows an opposite behavior compared to functional integration (see Materials and methods and Discussion). Dark- and light-blue lines superimposed over the time courses represent the intervals selected for statistical comparison of the mean values between deep and light anesthesia, respectively, and shaded areas indicate standard error of the mean (SEM). Deep and light intervals were statistically different for all measures (* indicates *p* < 0.05, ** stays for *p* < 0.01 and *** *p* < 0.001; see Results).

We induced a state of deep anesthesia in rats and recorded brain activity while the level of anesthesia gradually decreased (see Materials and methods; protocol scheme in Fig. 1A). Our first objective was to monitor BOLD signal changes over time that might correspond to different levels of anesthesia. To this aim, we first divided the continuous BOLD signals in 90% overlapping windows of 10 min and then analyzed, for each window, its variability for each region of interest (see Materials and methods). Supplementary Fig. S1A illustrates the variance of the BOLD signal of each single ROI, whereas the average across all areas is depicted in Fig. 2A. We observed a certain degree of heterogeneity depending on the ROI, but we also observed an overall increase in BOLD variability as the effect of anesthesia diminished in time. We then analyzed the time-varying changes of BOLD pairwise correlations over the entire duration of the protocol (see Materials and methods) and obtained, for each time window, the value of the correlations between different regions (referred to as functional connectivity (FC); Friston et al., 1993) and their average across all area pairs. We observed the footprint of a progressive modulation between different brain conditions, being characterized by a first drop of the correlations (due to the gradual descent toward deeper stages of anesthesia) followed by a gradual increase toward the end of the recording (see Figs. 2B and S1B in Supplementary material). As not all regions seemed to exhibit comparable correlation time courses, we also focused on the changes in pairwise correlation between the BOLD signal of each individual region and all other regions over sliding windows. This procedure gave us insights on the different functional connectivity pattern displayed by single cortical and subcortical areas as the effects of anesthesia gradually fade out over time (see examples of individual FC time courses of cortical and subcortical ROIs in Figs. S2 and S3 in Supplementary material). A complementary way to characterize the ongoing changes in FC across all brain areas is to focus on the variability in the distribution of all correlation coefficients over time (Figs. 2C and S1C in Supplementary material). We observed a change in the distribution of correlations, marked by a gradual spread toward higher positive values and a corresponding increase in its standard deviation (SD) as the effects of anesthesia diminished over time. All the obtained time-varying results showed a consistent tendency to increase over time, suggesting the presence of a modulation in the transition from deep toward light anesthesia (see Identification of Intervals corresponding to deeper and lighter states of anesthesia). One plausible explanation for such a pattern of observations was that the shape of the underlying BOLD distribution changed over time according to the level of anesthesia. To check for this possibility, we took as representative four ROIs whose BOLD signals showed the highest correlation during the light period (mPF and CC, bilaterally) and then compared the corresponding BOLD distributions obtained from the two levels of anesthesia. The distribution of the signal in the two periods had comparable shape, confirming the possibility to rule-out a spurious origin of the observed pattern of findings. We thus tested whether BOLD variance, pairwise correlation and its standard deviation were significantly different in the two intervals taken as representative of deep and light anesthesia. The mean values of both levels demonstrated that the light interval was characterized by higher values in all measures, being the pairwise correlation the most significant (BOLD variance: *p* = 0.0466; pairwise correlation: *p* = 0.0088; standard deviation of the correlation distribution: *p* = 0.0375, *one-sided paired t-tests*, *n* = 5). These results confirmed that deep anesthesia largely reduces brain functional connectivity, which is in turn gradually restored as the effect of anesthesia vanishes over time. Another evidence of the presence of distinguishable brain conditions was obtained measuring and comparing the degree of overall network synchronization in the mentioned intervals. We investigated this aspect by calculating the Kuramoto global order parameter χ (see Materials and methods), a measure that has been widely applied in neuroscience to investigate synchronization at different scales (Maistrenko et al., 2007; Cumin and Unsworth, 2007; Deco et al., 2009a; Breakspear et al., 2010; Cabral et al., 2011). Our results indicate that intrinsic BOLD fluctuations transiently synchronize across many ROIs either in deeper and lighter phases of anesthesia, nonetheless the mean synchronization during light was significantly higher than during deep anesthesia (with *p* = 0.0038, *one-sided paired t-test*, *n* = 5; see Fig. 2D).

### Functional integration and segregation during fading of anesthesia

Our results show that both correlation and overall networks' synchronization are globally reduced in deeper state of anesthesia. To further investigate the state-related changes in overall network properties, we quantified also functional integration and segregation (by means of the modularity index Q) across different levels of anesthesia (see Materials and methods). On one hand, we found that the progression from deep to light anesthesia implied a significant increase in functional integration, as shown when comparing the mean values obtained from the two intervals (*p* = 0.0067, *one-sided paired t-test*, *n* = 5; see Fig. 2E); on the other side, each relative increase (decrease) in functional integration over time was mirrored by a corresponding decrease (increase) in modularity. In addition, mean modularity was found to be significantly reduced in light anesthesia compared to deep (*p* = 0.00097, *one-sided paired t-test*, *n* = 5; see Fig. 2F). This pattern of finding is not unexpected, as the two indexes address complementary aspects of networks' organization: indeed, functional integration is related to the size of the largest connected component, whereas modularity, being a measure that quantifies network segregation, takes large values when a given graph can be partitioned in many scattered communities, which thus tend to be of relatively small size due to the finite size of the network.

### Lighter anesthesia is marked by the gradual emergence of functional networks

**Fig. 3:**
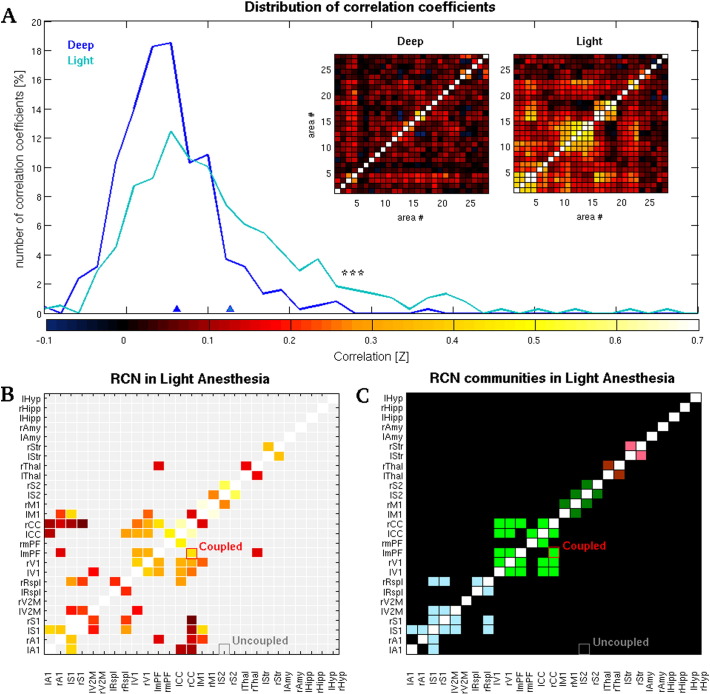
Different brain states are mirrored by different properties of the underlying functional networks. (A) Distribution of the correlation coefficients and corresponding average FC matrices obtained from deep and light intervals. The two distributions were statistically different (*** indicates *p* < *0.001*, see Results). Dark- and light-blue triangles indicate the means of the deep and light distributions, respectively. (B) Average matrix of the robust coupled nodes (RCNs; see Materials and methods) obtained from light anesthesia. The color-code quantifies the average correlation coefficient of those area pairs that were classified as RCNs. Area pairs that were not classified as RCNs are highlighted in light gray. (C) Community structure of the light RCNs, where nodes of the same colors indicate areas belonging to the same community (see Materials and methods). No community were detected in the deep anesthesia interval. In panels B and C, nodes that were selected as representative of “coupled” and “uncoupled” area pairs and used for further analysis are highlighted in red and gray, respectively.

**Fig. 4:**
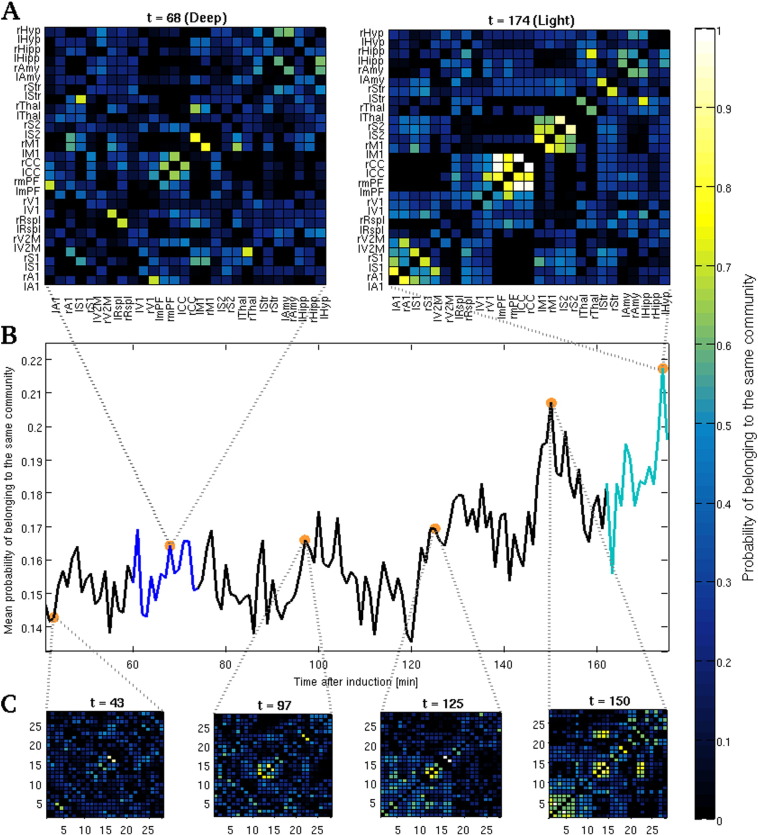
Functional networks' stability gradually increases while anesthesia progressively fades out. (A) Matrices of the ROIs pairwise *a posteriori* probability of belonging to the same functional community obtained from two sliding windows taken as representative of the deep (t = 68, left) and light (t = 174, right) anesthesia intervals. (B) Mean *a posteriori* probability of belonging to the same functional community over time. Superimposed dark- and light-blue lines indicate the deep and light anesthesia intervals, respectively. (C) Examples of the matrices of pairwise *a posteriori* probability that were used to compute the mean *a posteriori* probability depicted in panel B. During deeper phases of anesthesia the obtained community partitions are more variable both across iterations and animals, thus leading to sparser matrices and lower mean probabilities. Nonetheless, as the effects of anesthesia naturally decrease, BOLD fluctuations exhibit an increasing tendency to gradually stabilize in structured time-varying patterns of large-scale co-activations, as mirrored both by the increased mean probability of belonging to the same community as well as by the increasing consistency of the emerging communities. All *a posteriori* probabilities obtained for each sliding window were computed using all 10,000 iterations of the community detection algorithm over all animals (see Materials and methods).

FC matrices obtained from the two intervals showed distinct patterns and correlations' distributions, being the lighter period characterized by the presence of higher correlations and suggesting the emergence of structured patterns of brain activity (Fig. 3A). Statistical comparison confirmed that the average distribution of correlation values in the two conditions was significantly different (with asymptotic *p* = 7.5904·10^− 15^, *Kolmogorov*–*Smirnov test*). Due to the somewhat large variability among rats found in the correlation matrices of the light intervals, we selected only those area pairs that consistently showed robust correlations across subjects (RCNs; see Materials and methods). Collectively, the RCNs comprised less than 0.3% of all area pairs during deeper phases of anesthesia, indicating that the activity of the vast majority of areas during this state is generally less correlated with that of other areas, and less consistent across animals. Conversely, the number of RCNs showing consistent coupling between rats during lighter anesthesia was around 10% (Fig. 3B). The connectivity pattern among RCNs obtained from light anesthesia suggests that segregated networks of connected nodes emerge as a consequence of the gradual fading of anesthetic effect. To check for this possibility, we calculated whether the resulting RCNs obtained during light anesthesia could actually be partitioned into distinct functional modules by applying a well known community-detection algorithm (see Materials and methods). Consequently, five robust groups of functionally connected clusters of areas of different size were identified: The first RCNs community comprised the first auditory and somatosensory areas (A1, S1), secondary visual cortex (V2M) and the retrosplenial cortex (Rspl); the second comprised primary visual (V1), medial prefrontal (mPF) and cingulate (CC) cortices; and the third was composed by the primary motor (M1) and the secondary somatosensory (S2) cortices. The thalamus (Thal) and the striatum (Str) appeared to form two distinct functional modules of contralateral areas (Fig. 3C). The application of the same algorithm on the RCNs found during deeper phases of anesthesia returned no community structure. In addition, the time-varying matrices of the pairwise *a posteriori* probability of pertaining to the same functional community (see Materials and methods and Fig. 4) clearly indicate that the transition from deep to light anesthesia is marked by the gradual emergence of increasingly stabler patterns of structured activity. In fact, during deeper stages of anesthesia, the sets of community partitions that is possible to obtain across repetitions of the community detection algorithm are much less reliable, from the individual to the group level, than those obtained from light anesthesia. This indicates that deeper stages of anesthesia are dominated by a less structured pattern of co-activations than during lighter phases.

### FC time courses between areas belonging to the same functional networks

We next focused on the correlation time courses of two subsets of area pairs: (1) those that were consistently and highly correlated across all rats (*i.e.*, were previously identified as RCNs; see above and Materials and methods) during light anesthesia and, according to our community analysis, belonged to the same functional cluster (mPF and CC, henceforth referred to as “coupled” areas); and (2) those that showed no significant correlation (*p* ≥ 0.05) across all rats in light and deep anesthesia (A1 and S2, referred to as “uncoupled” areas). It should be noted that mPF and CC have recently been identified as part of the rat's default mode network (DMN, Lu et al., 2012). We analyzed the co-variation of the BOLD signals between the above mentioned ROIs including both ipsilateral and contralateral areas in order to control for possible differences in the co-activation pattern among areas belonging to the same or to the contralateral hemisphere. Comparisons of the mean correlation values obtained from deep and light intervals confirmed that there was no statistical difference between the two groups when considering uncoupled areas (A1-S2), whereas coupled ones (mPF-CC) showed a significant increase in correlation in the evolution from deep to light anesthesia (with *p* = 0.0117, *one-sided paired t-test*, *n* = 5, see Fig. 6A). In addition, it is worth to mention that FC, functional integration and mean synchronization suggest clear associations between each other (see Fig. S4 in Supplementary material). This is not surprising when considering that, for example, some of them are actually functions of other ones, or that can reflect a common mechanism. Nonetheless, by definition each of these measures quantifies different (even if related) properties, thus the fact that they exhibit linear association does not necessarily imply that they carry the same information.

### Fading of anesthesia is reflected by changes in neural coupling at specific frequencies

**Fig. 5:**
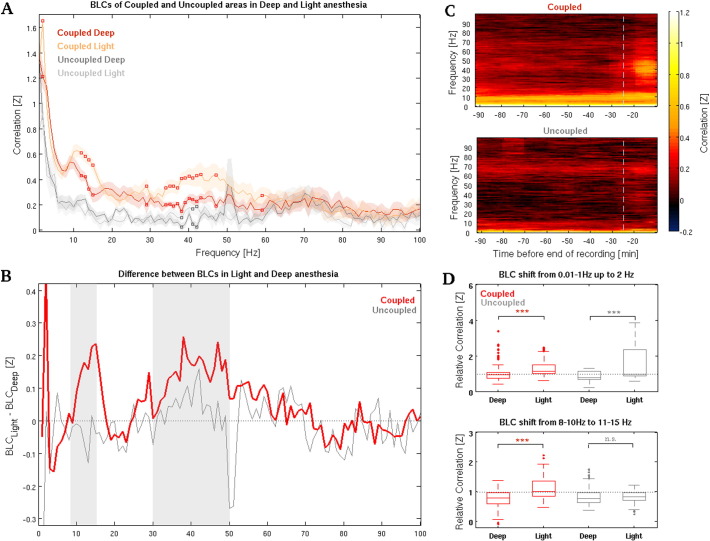
Band-limited correlations (BLCs) discriminate between network areas and different brain states at specific frequencies. (A) Average BLCs obtained from the two sets of areas. Empty squares mark BLCs that exhibited significantly higher values in light anesthesia compared to deep anesthesia within coupled (red squares, *n* = 10) and within uncoupled (gray squares, *n* = 6) area pairs (*p* < *0.05*; see Results). (B) Difference between 1 Hz-width BLCs obtained in light and deep anesthesia intervals was calculated separately for coupled (red line, *n* = 10) and uncoupled (gray line, *n* = 6) area pairs (see Materials and methods). Areas belonging to the same functional network (*i.e.*, coupled) selectively exhibit an increase in correlated oscillations at 8–15 Hz and 30–50 Hz (shaded areas) in the transition between deep and light anesthesia, whereas uncoupled areas do not show the same tendency. (C) Average BLC time courses of the two pairs of areas (see Materials and methods). Coupled areas (*top*) exhibit sustained correlations around 10 Hz that are preserved during all the recording session. These average BLC time courses are shown just for visualization purposes, as they resulted from individual recordings of slightly different length (see Materials and methods). Averages have been obtained aligning each recording to its end point and equalizing it with respect to the shortest one. This procedure does not allow showing the actual deep interval that was used for statistical comparison for each recording (see Fig. 1A). For this reason, only the onset of the light interval is shown, being represented by a light-blue dashed line. (D) Quantification of the BLCs peak shift in the transition from deep to light anesthesia in coupled and uncoupled area pairs. Average relative correlations (see Materials and methods) were statistically different in all cases except for the shift from 8–10 to 11–15 Hz in uncoupled areas. *** indicates *p* < 0.001, whereas *n.s.* stands for ‘*non significant*’ (see Results).

We thus selected the aforementioned coupled and uncoupled areas for further LFP recordings in order to determine the specificity of oscillatory patterns connecting distinct area pairs during the gradual fading of anesthesia. To that end we simultaneously recorded LFPs from mPF, CC, A1 and S2 while using the same anesthesia protocol we had used in fMRI experiments. Comparison of the power spectra between deep and light intervals confirmed that ketamine–medetomidine anesthesia induced a modulation in the oscillatory activity. This modulation was characterized by a decrease in power at frequencies higher than 30 Hz in all areas and also by an increase in power in frequencies around α in mPF and CC (*p* < 0.05, *paired t-test*, see S5 in Supplementary material). We then computed BLCs (see Materials and methods) of the two different area pairs obtained from deep and light intervals and statistically compared the mean values within coupled (mPF-CC, *n* = 10) and uncoupled (A1-S2, *n* = 6) areas. Whereas for uncoupled areas the BLCs between different states differed only slightly, coupled areas showed a significant increase in BLCs during light anesthesia at different frequencies (*p* < *0.05*, *one-sided paired t-test*, *n* = 10, see Fig. 5A). This increase appeared selectively at 8–15 Hz and in the 30–50 Hz range. In addition to this, the non-overlap of the standard mean error intervals shows that coupled areas exhibit higher correlations than uncoupled ones in both anesthesia conditions continuously from 2 to 50 Hz (see Fig. 5A). To further investigate this fact, we compared the correlations' distribution of coupled and uncoupled areas both during deep and light anesthesia for the two above-mentioned frequency ranges (8–15 Hz and 30–50 Hz), confirming that coupled areas showed significantly greater correlations in both comparisons (all *p* < 0.001, *Wilcoxon rank sum test*, coupled *n* = 10, uncoupled *n* = 6). The frequency-specific increase from deep to light anesthesia seen in coupled areas became evident when the BLCs obtained during deep anesthesia were subtracted from that obtained during light anesthesia, as depicted in Fig. 5B (see Materials and methods). These findings not only demonstrate that areas belonging to the same functional network correlate at specific frequencies more than areas that do not participate in the same network even during deeper stages of anesthesia, but they also indicate that coupled areas exhibit a clear net increase in coupling in these frequency ranges (8–15 Hz and 30–50 Hz) during the progression to lighter states of anesthesia. Subsequently, in order to analyze the evolution of neuronal coupling at different frequencies over time, we divided the original preprocessed LFP signal in sliding windows (as done for fMRI) and then computed the BLCs in each window (see Materials and methods). The resulting band-limited time courses confirmed that it is possible to distinguish between coupled and uncoupled nodes even during deeper phases of anesthesia, given that areas participating in the same network showed stronger correlations at approximately 10 Hz (see Fig. 5C).

### State- and network-related shifts to higher frequencies

The evolution to light anesthesia in coupled areas was marked by a gradual shift toward higher frequencies of the correlation peaks at approximately 1 Hz, and another around 10 Hz. This fact prompted a more detailed investigation. We quantified these possible state-related shifts to higher frequencies calculating the relative correlation between higher and lower components of the frequency ranges of interest (see Materials and methods). Statistical comparison of the mean relative BLCs in deep and light intervals showed that the shift from < 1 Hz up to 2 Hz significantly accounted for the progression from deep to light anesthesia in both area pairs (coupled: *p* = 1.7288 · 10^− 8^, *n* = 10; uncoupled: *p* = 5.335 · 10^− 9^, *n* = 6; *paired Wilcoxon signed rank test*; see Fig. 5D *top*). On the other hand, the increase in relative correlation from 8–10 to 11–15 Hz from deep to light anesthesia was significant only in areas that belonged to the same functional network (coupled: *p* = 4.4988 · 10^− 17^, *n* = 10; uncoupled: *p* = 0.8327, *n* = 6; *paired Wilcoxon signed rank test*; see Fig. 5D *bottom*). These findings suggest that the BLC shift from 8–10 to 11–15 Hz observed in the transition from deep to light anesthesia could be specific to areas participating in common functional networks.

### Connectivity time courses in fMRI and LFPs exhibit similar tendencies

**Fig. 6:**
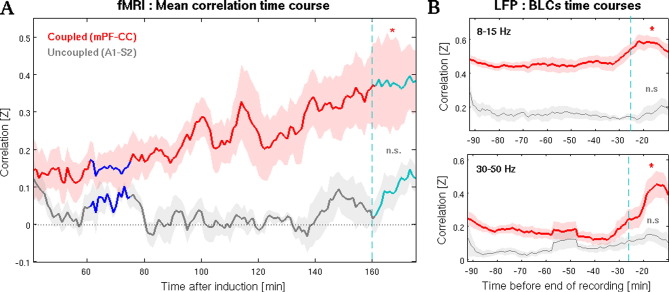
BOLD coherent fluctuations and BLCs of specific frequency ranges exhibit comparable time courses. (A) Mean BOLD correlation time courses obtained averaging the activity of only those ROIs that were selected as being representative of areas that belong to the same (coupled, red) or to different (uncoupled, gray) functional networks (see Materials and methods). (B) Examples of average BLCs time courses for two different frequency ranges for the two sets of area pairs (see Materials and methods). Light-blue, dashed lines in panels A and B indicate the onset of the light interval, whereas * indicate significance (p < 0.05) of the statistical comparison deep *versus* light and *n.s.* indicates “not significant” (see Results). Due to the different duration of individual LFP recordings, deep intervals used for statistical comparisons are not shown in panels B (see legend of Fig. 5).

The obtained BLC time courses (Fig. 5C) indicated that the progression to light anesthesia is characterized by an increase in correlation that is specific for the above-mentioned frequency ranges (8–15 Hz and 30–50 Hz), and can account for differences between areas belonging to the same or to different functional networks. Not surprisingly, statistical comparisons of the mean correlations of the BLC time courses obtained from both periods confirmed that only areas pertaining to the same functional network exhibit significant increase in correlations in the 8–15 Hz (α) and 30–50 Hz (low γ) bands during the transition from one state to the other as compared to other frequency bands (α band: *p* = 0.0317; low γ band: *p* = 0.0287; *one-sided paired t-test*, *n* = 10; see Fig. 6B). Qualitative comparison of the BLCs over time between all area pairs simultaneously recorded in LFPs experiments and the corresponding FC time courses obtained from the same ROIs (mPF-CC and A1-S2) in imaging experiments outline the possible existence of a relationship between brain correlated fluctuations as measured with BOLD signal and coupled neural oscillation, especially in the γ range (see Figs. 6 and S6 in the Supplementary material).

## Discussion

Neurons form relatively stable structural connections in the brain, but at the same time they participate in functional networks that change over time according to the brain state. Despite its relevance, few studies have focused on the ongoing changes between different brain states (Martuzzi et al., 2010; Tang et al., 2012; Lewis et al., 2012). To our knowledge, none has addressed this question by comparing measurements obtained with imaging techniques and intracortical recordings in order to investigate their consistency. The aim of the present work was to identify how the gradual evolution from deeper to lighter phases of anesthesia modulates the connectivity patterns between different areas at the macro- (fMRI) and the mesoscopic (LFPs) levels, and to determine whether such coupling motifs could distinguish between areas belonging to the same or to distinct functional networks. To that end, we compared results separately obtained with fMRI and LFPs using a common experimental protocol. Having deeply anesthetized the animal, we recorded rat brain activity while the level of anesthesia progressively decreased over time. As expected, our findings indicated that the induction of a deep state of anesthesia has dramatic effects on brain functionality. Indeed, the most profound phase of anesthesia is characterized by a significant reduction in the variability of intrinsic BOLD fluctuations and their overall low-frequency synchronization. These phenomena can explain the observed weakening of long-range correlations between different regions and the transient breakdown of those differentiated networks that have previously been found in rats during light anesthesia (Pawela et al., 2008) and waking rest (Liang et al., 2011). Our findings are in agreement with the proposal that the induction of anesthesia is related with a reduction in the repertoire of distinguishable brain states (Alkire et al., 2008; Hudetz et al., 2014), as the decrease in variability of both the BOLD signal and the correlation distribution leads the system toward more uniform, stereotypical dynamics (Deco et al., 2009b). It is well known that the resting brain exhibits small-world network properties (Sporns and Zwi, 2004; Bassett and Bullmore, 2006), where a large number of densely clusterized local networks performing highly segregated computations are linked together by a relatively small number of connections, resulting in a complex structure of hierarchically nested networks (Meunier et al., 2009; Sporns, 2013). This complex architecture has been theoretically linked with efficient information processing and propagation, enabling to rapidly integrate the outputs from different specialized local networks (Watts and Strogatz, 1998; Latora and Marchiori, 2001). Our results suggest that during deeper stages of anesthesia these functional connections between communities get dramatically weakened. The resulting framework of scattered local connectivity is thus mirrored by an increase in functional segregation, and a corresponding reduction in integration. In this scenario, deep phases of anesthesia are characterized by a massive destructuration of the complex large-scale pattern of co-activations seen during light anesthesia (Pawela et al., 2008; Lu et al., 2012) and waking rest (Liang et al., 2011; Zhang et al., 2010). As the effect of anesthesia vanished, the coupling across distant brain regions is gradually restored, leading to the progressive flourishing of distinct functional networks, as was also indicated by the corresponding rise in functional integration. Taken together, these results indicated that the gradual emergence from anesthesia is marked by an increase in differentiated and structured large-scale brain activity. Indeed, during fading of anesthesia, intrinsic activity of different subsets of areas start to cluster, uncovering the presence of a non homogeneous correlation structure which reveals itself as the animal approach the wake state, and that likely reflects the progressive and dynamical emergence of different functional networks. After induction of a deep state of anesthesia, the precisely organized pattern of reliable co-activations that have been found to characterize resting brain activity undergo a dramatic reduction in their overall inter-areal synchronization, which in turn lead to the partial fragmentation of the large-scale brain networks into an highly variable mosaic of smaller clusters of areas exhibiting transients of weakly correlated activity. It should be emphasized that even if small correlation coefficients indicate weaker statistical dependence, it does not mean that the vast anesthetic-induced net decrease in pairwise correlation would simply result in pure random brain activations: deeper stages of anesthesia seem to be rather described by a more volatile scenario where small subsets of areas belonging to common networks show reduced correlated fluctuations of the BOLD signal and present decreased probability of exhibit collective correlated oscillations, which in turn could explain the transitory breakdown of large-scale functional networks. Our *in vivo* results support this view, as they provide evidences for the preserved presence, even during profound phases of anesthesia, of a functional backbone consisting of highly correlated oscillations in the α band that maintained linked to each other areas that belong to the same network. During the progressive fading of anesthesia, the rather scattered fragments of functional clusters seen during deeper stages exhibit a tendency to bind themselves together in an increasingly stabler fashion, giving rise to the gradual emergence of time-varying functional networks. It is worth to mention that subcortical structures exhibited a functional connectivity pattern that was very different compared to that of cortical regions, both quantitatively and qualitatively. In fact, subcortical areas displayed overall lower correlations with other areas than cortical ones, together with a less clear footprint of functional clusterization. Another noteworthy feature is the temporal pattern of co-activations within and between the amydgalas and the hypothalami, which exhibited a peak during the deep phase and then decreased as the effect of anesthesia gradually vanishes, whereas most of the cortical areas (and to some extent the thalami) present increasing functional connectivity over time. These observations, together with recent results showing that local inactivations of wake-active subcortical areas enhance general anesthesia (Leung et al., 2014) suggest that cortical and subcortical structures react to anesthetic in a different fashion, being cortical regions the first ones to recover from anesthetic-induced inactivations compared to subcortical ones. Interestingly, some of the regions that, on a group level, first showed correlated patterns of BOLD fluctuations while decreasing the level of anesthesia are in fact part of the rat's DMN (Lu et al., 2012), namely mPF, CC, A2, V2M and retrosplenial (Rspl) cortex. Other areas were mainly primary cortices (M1, S1, V1), followed by S2, the thalami and the striatum. The DMN, first isolated in humans (Raichle et al., 2001), has been linked to functions ranging from conceptual processing (Binder et al., 1999) to self-referential functions (Buckner and Carroll, 2007; Buckner et al., 2008) and awareness (Horovitz et al., 2009), and has been found to be abnormally connected in neuropsychiatric disorders (Whitfield-Gabrieli and Ford, 2012). Interestingly, the DMN has also been found in lightly anesthetized humans (Greicius et al., 2008), chimpanzees (Rilling et al., 2007) macaques (Vincent et al., 2007) and rodents (Lu et al., 2012; Stafford et al., 2014), thus suggesting its evolutionary relevance and persistence in different states, such as during light sedation and the early stages of non-REM sleep (Sämann et al., 2011). Lu and colleagues (Lu et al., 2012) showed that the rat DMN is divided into two modules: one parietal and the other temporo-frontal. Notably, we found that the activity of mPF and CC, core areas of the DMN's temporo-frontal module, was tightly related, whereas V2M and Rspl both participated in another module. Collectively, these evidences indicated that the DMN is one of the brain networks that first emerges in the transition from deep to light anesthesia. Such a finding is not unexpected, given the vast anatomical connectivity between DMN regions and other cortical and subcortical areas (Lu et al., 2012) that leads to the convergence of incoming activity from many other different regions. Indeed, in humans it has been found that the posterior part of the DMN is one of the most connected areas of the brain (Hagmann et al., 2008). Therefore, it is conceivable that this anatomical confluence of connections from distant areas, together with the dense connectivity within the DMN, could produce amplification in network synchronization, thus making the DMN one of the first networks to emerge in the transition from a state of decreased activity, as exemplified by deep anesthesia. These results are coherent with the finding that, in humans, the physiological, induced or abnormal reductions in the level of conscious awareness are linked with low metabolism in the precuneus and in the posterior cingulate cortex (Laureys et al., 2004), which is structurally homologous to the rat's retrosplenial cortex (Lu et al., 2012). Decoupling of the anterior and posterior components of the DMN has been found to characterize the descent to slow-wave sleep (Sämann et al., 2011) as well as Propofol-induced anesthesia (Boveroux et al., 2010), and decreased activity and connectivity between these regions are also seen in states of unconstrained cognition induced by psychedelic agents like psylocibin (Carhart-Harris et al., 2012). Taken together, all these evidences suggest that the overall integrity and coherency of DMN activity plays an important role in the modulation of either the state and the content of consciousness. Another noteworthy finding is that, in our data, the activity of the left and right thalami started to be robustly correlated only during the light phase of anesthesia (Fig. 3C; see Results). This subcortical structure is the principal gateway of sensory information flowing from the periphery to the cortex (Steriade et al., 1993), and anesthesia-induced deactivations of the thalamus are commonly found in imaging studies (Franks, 2008). Thus the observation that the thalamic nuclei started to entrain in correlated co-activations during light anesthesia could be interpreted as a preliminary step for the gradual recovery from anesthesia-induced non-responsiveness. Our results show that different regions exhibit different dynamics in recovering from deep phases of anesthesia, thus leading to a gradual patterns of increasing co-activations which likely end up in the progressive reconfiguration of resting state networks; indeed, in the transition from deep to lighter states of anesthesia we observed that the two principal cores of the rat's DMN start to show a strong and consistent pattern of within-core correlations, but our analysis indicated that the collective activity of the two main branches of the DMN seems to be still partially decoupled at this stage of anesthesia, thus suggesting that full network recovery from deep anesthesia fragmentation is a rather continuous but slow process, and in fact we couldn't observe full DMN recovery in our imaging experiments. On a mesoscopic scale, our LFPs results indicate a specific connectional footprint marking the transition from one state to the other. In fact, we found that local neural coupling between regions belonging to the same functional network seems to be preserved even during profound phases of anesthesia. In fact, these areas maintain larger correlated oscillations up to 50 Hz, exhibiting a peak in the α range. Areas belonging to different networks did not show such a strong and consistent correlation peak. This finding reveals the presence of a frequency-specific functional bridge that is preserved even during deeper states of anesthesia in areas belonging to the same functional network, and likely endowed by a consistent underlying anatomical connectivity. As the animal approaches lighter stages of anesthesia, the frequency-coupling scenario became richer. Correlated oscillations in the α band shift toward higher frequencies, up to 15 Hz. It should be noted that recent MEG studies in resting humans have found that the correlations between areas pertaining to the same networks exhibit a peak between 10 and 20 Hz (Brookes et al., 2011; de Pasquale et al., 2010; Hipp et al., 2012), and further computational evidence supports those results (Cabral et al., 2014b). More specifically, correlations between DMN areas have been shown to be mostly between 8 and 13 Hz in humans (Brookes et al., 2011). Another feature related to the evolution toward light anesthesia is the appearance of highly correlated oscillations in the low γ range (30–50 Hz), which is particularly strong between areas belonging to the same functional network. It has been proposed that effective communication between neuronal ensembles is dependent on their ability to oscillate synchronously (Singer, 1999; Fries, 2005), and particularly the synchronization in the γ range is thought to serve as a core computational mechanism between local cortical networks (Fries, 2009). One could thus speculate that the significant drop of correlated γ oscillations seen during the most profound phases of anesthesia could mirror the cortex' inability of performing finely integrated neural computations; this phenomenon, together with the networks' fragmentation characterizing deep anesthesia, suggests some interesting similarities with the breakdown of cortical effective connectivity seen during NREM sleep (Massimini et al., 2005). Deep anesthesia and NREM sleep both are states dominated by local instead of propagating large-scale brain activity, and our results showed that deep anesthesia is indeed characterized by a significant reduction in functional integration. These evidences are in agreement with the hypothesis that the apparent absence of consciousness seen during deep anesthesia and NREM sleep could be linked with weakened or interrupted brain's ability to integrate information (Alkire et al., 2008; Tononi and Massimini, 2008). We believe that taken together, our findings could indicate that one of the global effects of anesthesia is that of reducing the overall dynamical complexity, as also suggested by findings indicating a decrease in EEG signal complexity in deep anesthesia (Bruhn et al., 2000; Zhang et al., 2001; Ferenets et al., 2007). Finally, it should be noted that our imaging and *in vivo* results exhibited an interesting degree of qualitative consistency, as both point toward an increase in correlated activity during the progressive emergence from deep anesthesia. In particular, LFPs band-limited correlations in the α (8–15 Hz) and especially in the low γ (30–50 Hz) ranges showed the highest similarity to the mean correlation time courses obtained from imaging experiments (Figs. 6 and S6 in S*upplementary Material*). This observation is in accordance with the evidence that increases in the BOLD signal reflect increases in neural activity (Logothetis et al., 2001; Viswanathan and Freeman, 2007), and that the power of LFPs in the mentioned frequencies conveys information about the timing and the amplitude of the hemodynamic signal (Kayser et al., 2004; Niessing et al., 2005; Schölvinck et al., 2010; Pan et al., 2011; Magri et al., 2012). Future studies directly investigating the relationship between correlated BOLD fluctuations and BLCs at specific frequencies will be highly informative in shedding light on the neurophysiological basis of BOLD functional connectivity.

### Limitations

The present study has some intrinsic limitations, thus the interpretation of reported results have to be done cautiously. We induced a profound state of anesthesia using a mixture of ketamine and medetomidine, a combination widely used to anesthetize animals. In fact, ketamine alone produces deep sedation but not surgical anesthesia, which is achieved combining it with medetomidine or xylazine, which supplements ketamine's effect with analgesic properties, muscle relaxation, and sedation. The two drugs have different and specific neurophysiological mechanisms and metabolic properties (ketamine is an NMDA antagonist, whereas medetomidine and xylazine are α-2 agonists, see Brown et al., 2010, 2011), thus the state of anesthesia and the recovery pattern they induce may not be generalized to those determined by other anesthetic agents. It should also be noted that, even though the animals had been treated under the same procedure conditions, other variables cannot be controlled, such as the stress of the animal at the moment of anesthesia induction, the body-fat percentage or the metabolic rate of each animal. The lack of control over these factors produces an individual variability in the time course of the anesthesia across different subjects. In addition to this, the lack of a direct assessment of the ongoing anesthetic concentration at any given time imposed us to use a *post-hoc* approach to find those protocol intervals that simultaneously showed significant differences in many measures across rats, and that most likely represented different anesthetic depths. This in turn ensured that the properties of the signals recorded from the two chosen intervals were actually different, but did not imply that the activity recorded in the deep (light) interval represented exactly the same anesthetic depth among animals. Nonetheless, the chosen intervals largely account for the individual pharmacokinetics of the two drugs (Sinclair, 2003; Brown et al., 2011; Quibell et al., 2011), and can thus be taken as representative of deeper and lighter phases of anesthesia. Despite this limitations, and given that fMRI and LFPs weren't recorded simultaneously from the same animal and had slightly different durations, the overall consistency of the present results firmly demonstrates the differential modulation of intrinsic brain activity induced by naturally decreasing levels of anesthesia.

## Conclusions

The progressive emergence from deep anesthesia is characterized by an increase in correlated large-scale low-frequency fluctuations, as well as by an enhancement in the local coupling of band-limited oscillations between areas participating to the same functional network. On the other hand, more profound phases of anesthesia are marked by a decrease in differentiated activity. Progressive fading of anesthesia is mirrored by the gradual flourishing of highly organized spontaneous brain activity, being the default-mode network one of the first networks to emerge. Nonetheless, we observed that local frequency-specific connectivity between areas participating to the same functional networks is preserved also during deeper phases of anesthesia, indicating a partial maintenance of brain functional organization even during states of deep sedation.

## Author contributions

R.G.B., N.T.C., M.R.M., M.V.S.V. and G.D. designed the research; N.T.C. and M.R.M. performed the research; R.G.B. analyzed the data; and R.G.B., N.T.C., M.R.M., M.V.S.V. and G.D. wrote the paper. R.G.B., N.T.C. and M.R.M. equally contributed to the paper.
