# Supplementary material for: Gradual emergence of spontaneous correlated brain activity during fading of general anesthesia in rats: Evidences from fMRI and local field potentials
Source: Neuroimage. 2015 Jul 1;114:185–98. doi: 10.1016/j.neuroimage.2015.03.037 (PMC4461308; doi:10.1016/j.neuroimage.2015.03.037)
Supplement: Supplementary file 1 — Supplementary material. [file mmc1.pdf]

# SUPPLEMENTARY MATERIAL

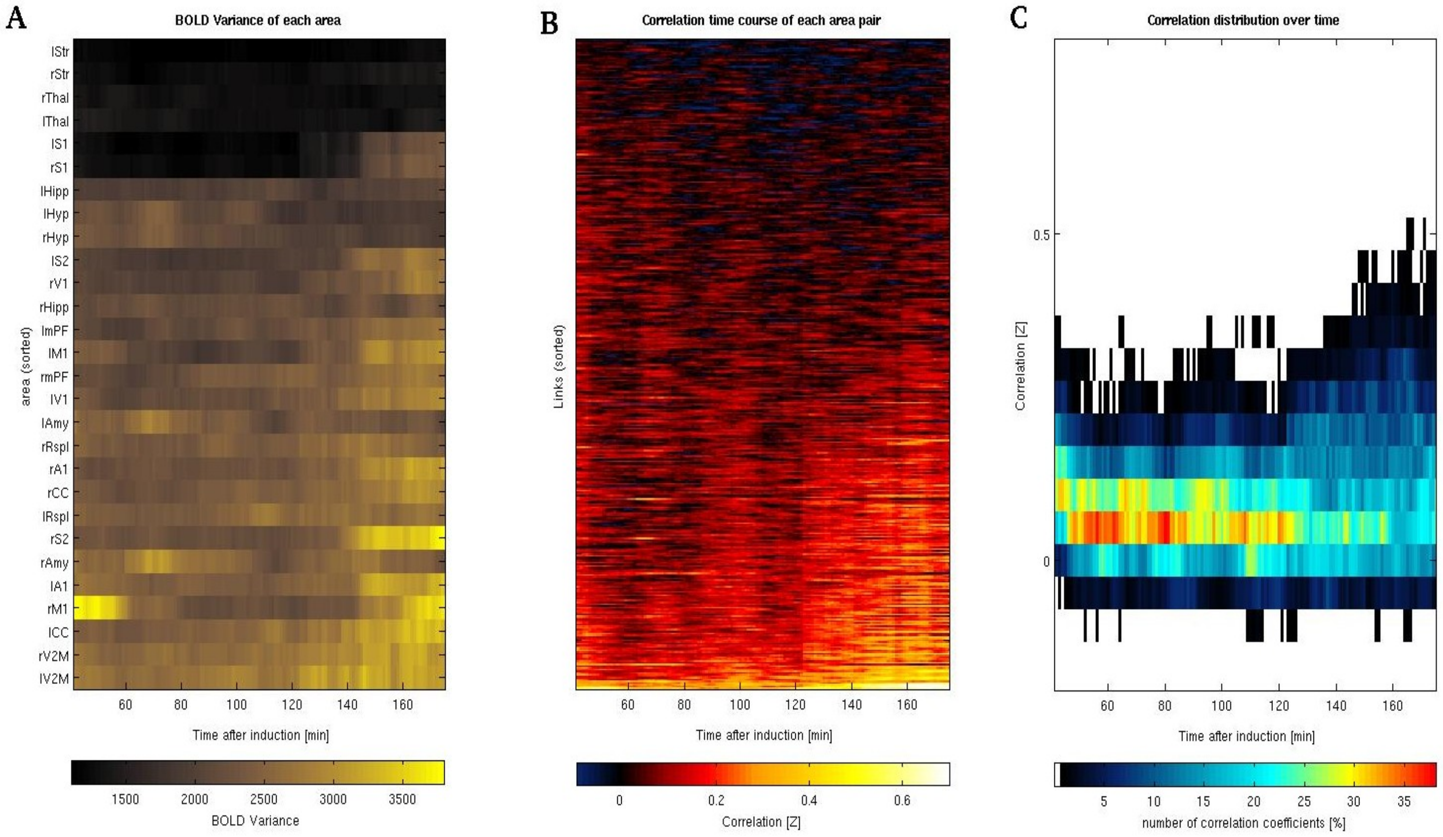


**Figure S1. fMRI: Gradual changes in signal variability and FC as anesthesia naturally fades out.**

(A) BOLD variance over time in each area. All areas has been sorted depending on their mean value across time.

(B) Correlation time course of each link (*i.e.,* each pair of areas) under different levels of anesthesia. Links has been sorted depending on their mean value across time. Pearson's linear correlations were converted to the corresponding Fisher's *z-score* (see *Materials and Methods*).

(C) Distribution of all correlation coefficients over time. First phases of anesthesia are characterized by more symmetrical distributions centered around low correlation values, whereas while the effect of anesthesia progressively decreases, the percentage of links showing higher correlations raises.

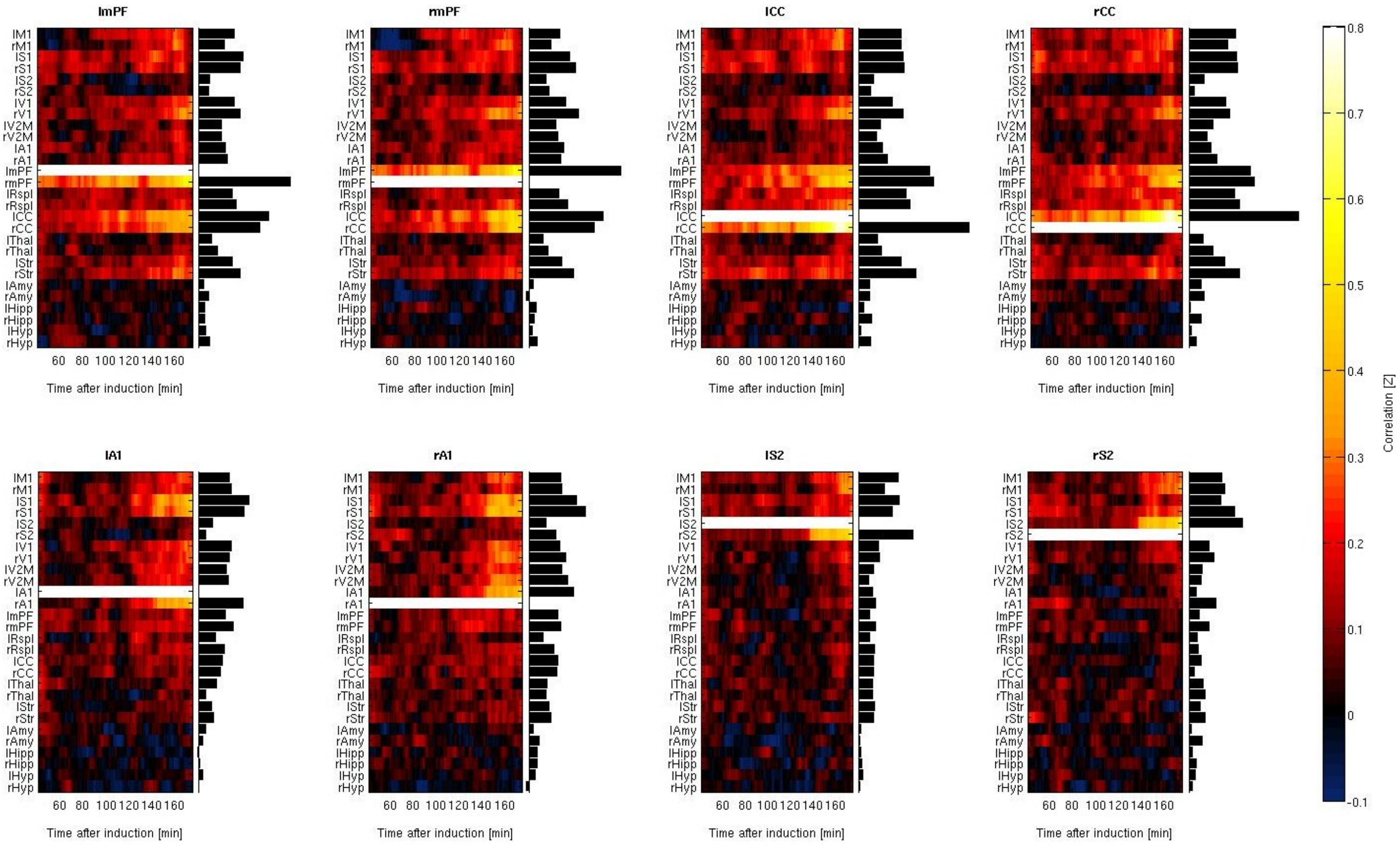


**Figure S2. Individual FC time courses of a subset of cortical ROIs.**

Each panel depicts the functional connectivity time course of a given cortical area with all other areas, computed from 90% overlapping sliding windows of 10 minutes. The horizontal bars on the right of each panel represent the mean pairwise correlation between the ROI and all other areas calculated across all the recording, and emphasize which are the preferred subset of regions that exhibit the highest correlations with a given area over all protocol time. A first feature that is possible to appreciate is that contralateral ROIs show increased correlation as the effect of anesthesia gradually vanishes over time, being its value among the highest ones, where not the highest itself. In addition, these analysis strongly suggest the presence of an underlying correlation structure, which become clearer as anesthetic effects gradually fades out over time. Indeed, cortical regions exhibit the tendency to cluster preferentially with different subsets of other regions, as exemplified by mPF and CC, which seem to take part to the same functional cluster (top panels), and by A1 with S1 and V2M (bottom-left panels) and by S2 with primary sensory (S1) and motor (M1) cortices (bottom-right panels).

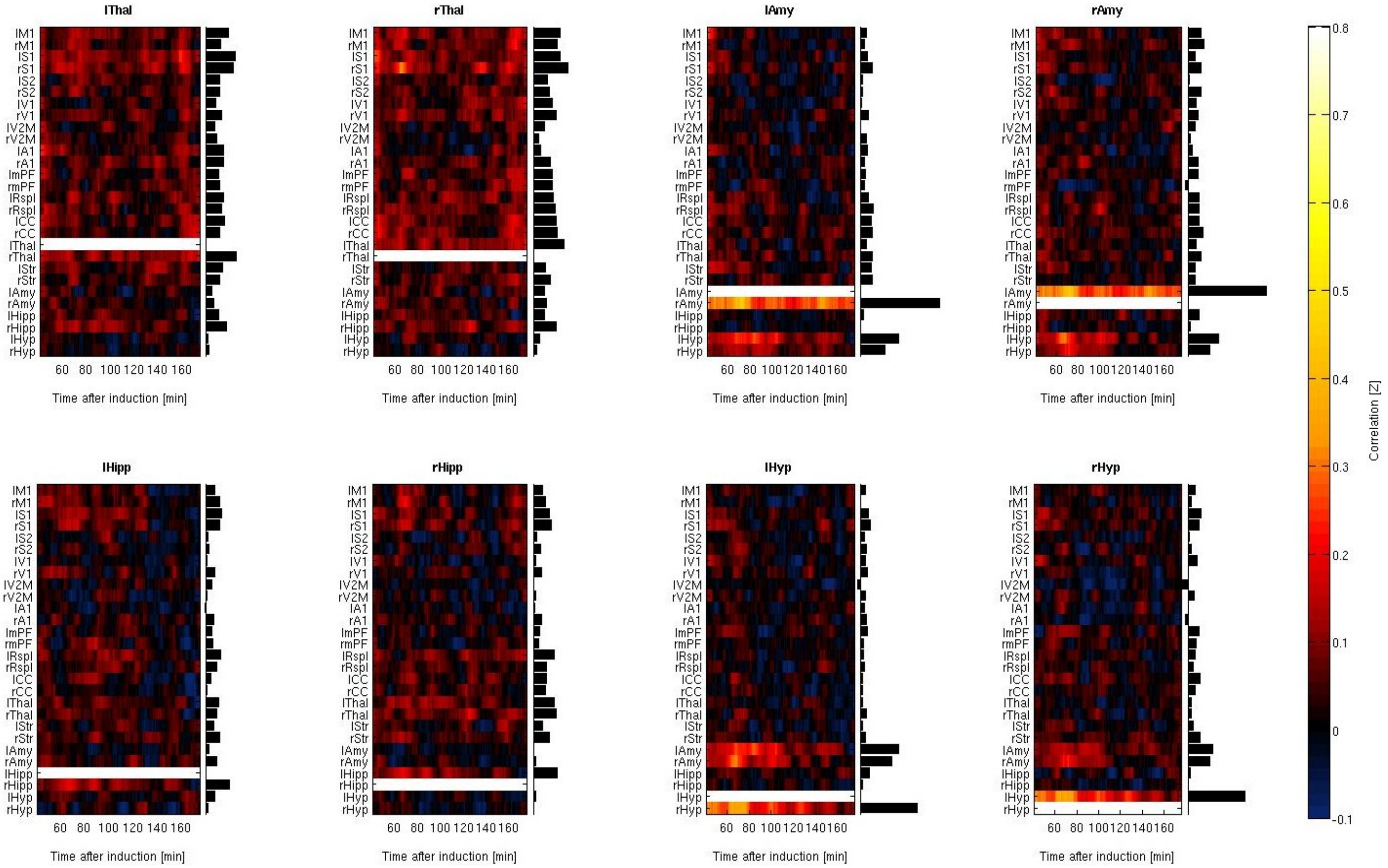


**Figure S3. Individual FC time courses of a subset of subcortical areas.**

Each panel depicts the functional connectivity time course of a given subcortical area with all other areas, computed from 90% overlapping sliding windows of 10 minutes. The horizontal bars on the right of each panel represent the mean pairwise correlation between the ROI and all other areas calculated across all the recording, and emphasize which are the preferred subset of regions that exhibit the highest correlations with a given area over all protocol time. Subcortical areas tend to show overall lower correlations with other areas than cortical ones. Interesting is the temporal pattern of coactivations within and between the amydgalas and the hypothalami (top-right and bottom-right panels), which (after a first increase during deeper stages of aneshesia) is decreasing, whereas most of cortical areas (see figure S2) and to some extent the thalami (top-left panels) exhibit increasing functional connectivity over time.

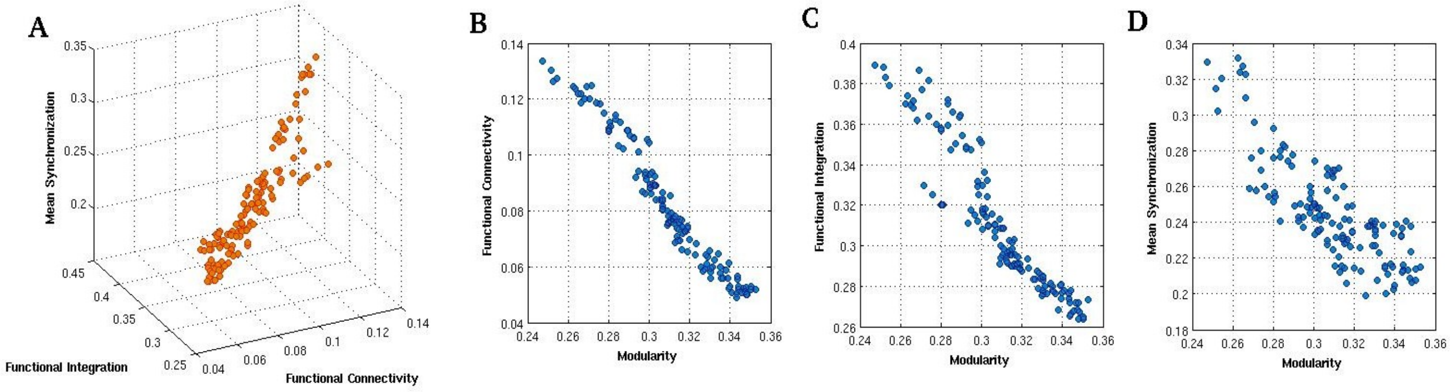


**Figure S4. Scatterplots of variables measuring different aspects of functional dynamics.**

(A) Three dimensional scatterplot of the FC, functional integration and mean synchronization time courses. It is possible to see that, as expected, the three variables are highly correlated. Pairwise correlation between FC and functional integration is 0.9596, that between FC and mean synchronization 0.8069 whereas between functional integration and mean synchronization is 0.8009.

(B) The relationship between Modularity Q with the three measures plotted in panel A confirms that this metric conveys complementary information with respect to the others. Pairwise correlation between Modularity and FC is in fact - 0.9775.

(C) Correlation between Modularity and funcional integration is – 0.9370.

(D) Correlation between Modularity and mean synchronization is – 0.8182.

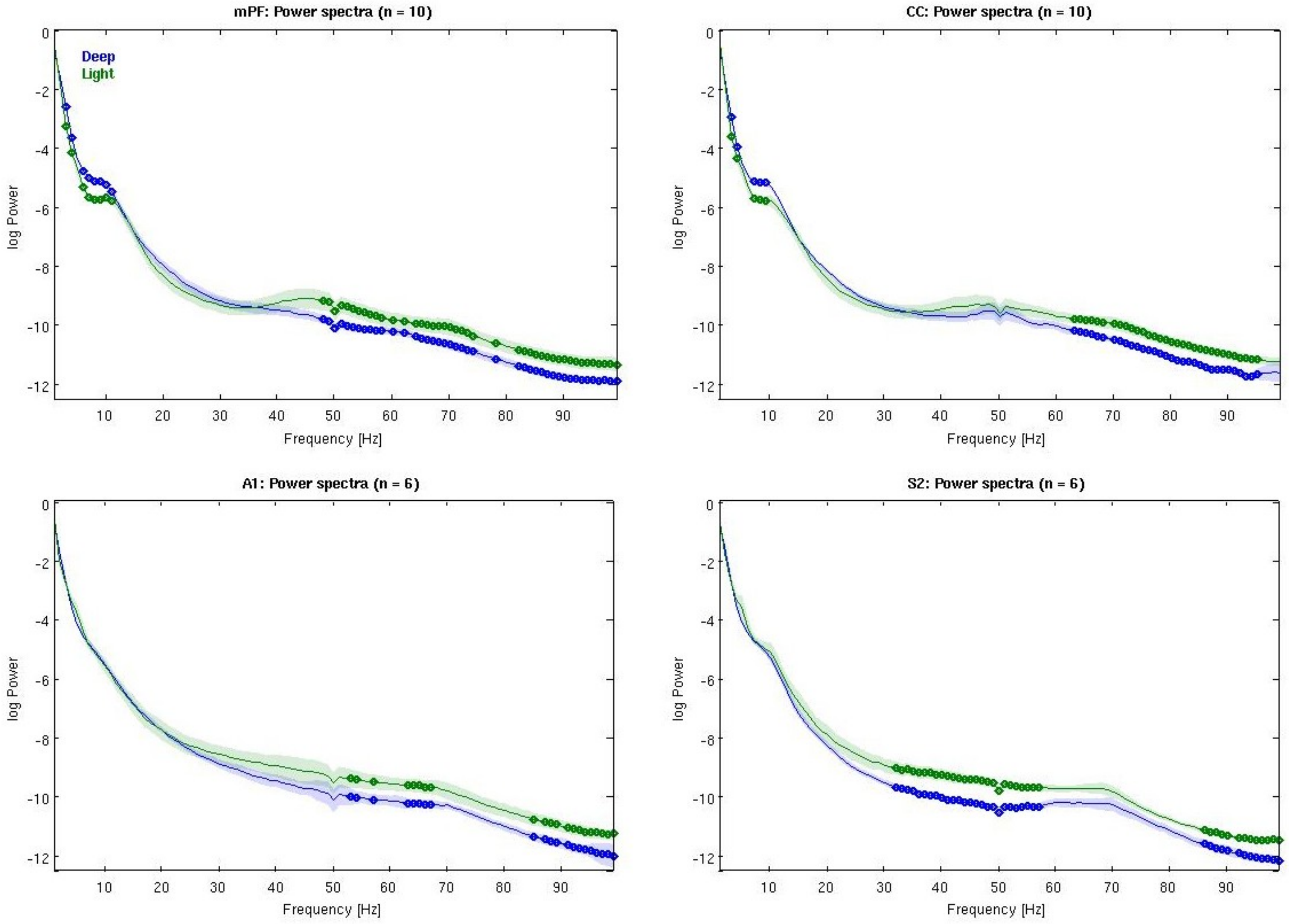


**Figure S5. Ketamine/medetomidine modulation of the power spectra in deep and light intervals.**

Empty rhombuses indicate individual frequencies whose power were significantly different in the two intervals recorded in *in vivo* experiments ($p < 0.05$, *paired t-test*). It is possible to appreciate that during deep anesthesia the power of oscillations tend to be somehow reduced over all the spectrum above 25-30 Hz in all four recorded areas, whereas power in the α range (~10 Hz) is increased for DMN areas (mPF and CC). It should be noted that, compared to A1 and S2, mPF and CC presented a more prominent peak at around 10 Hz both in deep and light anesthesia. Shaded areas represent the standard error of the mean.

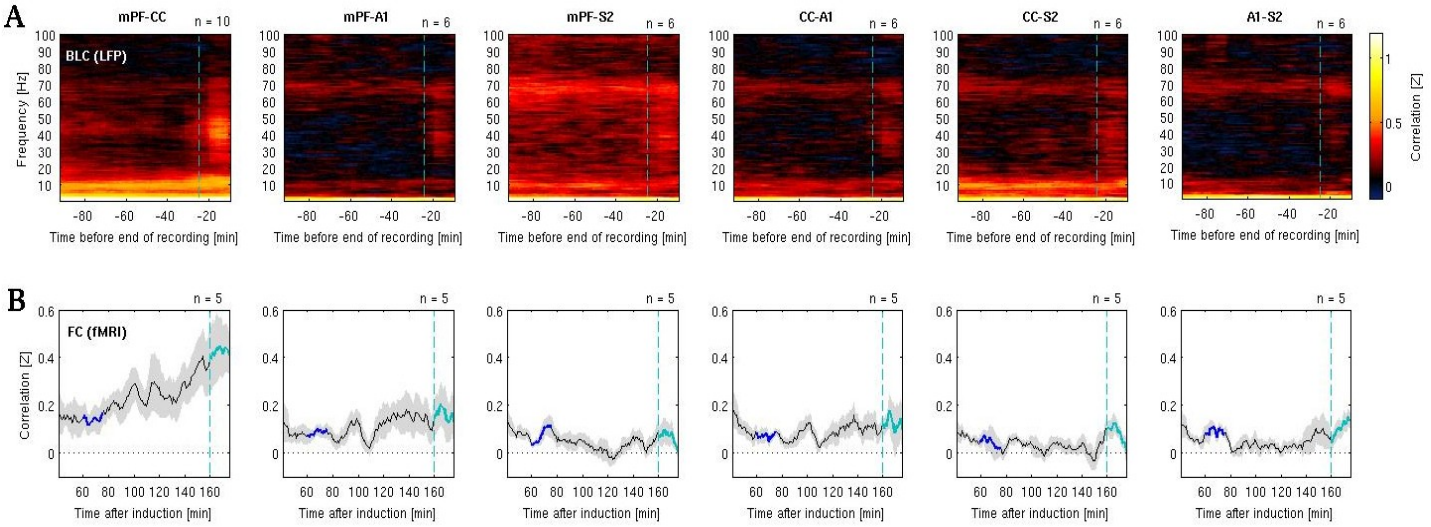


**Figure S6. BLC time courses of all area pairs simultaneously recorded in LFP experiments and BOLD FC time courses of the corresponding ROIs.**

(A) Average band-limited correlations (BLC) over time. Note that individual LFP recordings were slightly different in duration (see Figure 1A). Indeed, the present panels where plotted just for visualization purposes, as it was not possible to highlight an unique "deep" interval. In fact, before averaging the BLC were aligned to the end point and then equalized with respect to the shortest one. For this reason, only the interval corresponding to light anesthesia is marked, starting in correspondence of the dashed light-blue line and until the end of the recording (see *Materials and Methods*).

(B) Correlation time courses of the corresponding pairs of ROIs obtained from separate fMRI scans. Dark- and light-blue lines superimposed over the time courses in panels B represent deep and light intervals , respectively.

Interpretations of the possible relationship between BLC and FC time courses are not straightforward. In fact, imaging and *in vivo* experiments were not simultaneous, made use of different rats and had different durations, factors that imposed methodological limitations in performing a direct and reliable measurement of their association. Nonetheless, it is possible to qualitatively appreciate a number of features, both within and between the two different recording techniques. First, BLCs tend to exhibit a relative increase during the light interval at around 30-50 Hz (low-γ) in virtually all cases, the magnitude of this increase being the factor that apparently distinguished between mPF-CC and the other pairs of areas. Second, they also showed a rather persistent presence of correlated oscillations around 10 Hz (α) and another at around 70 Hz (mid-γ), both of which seemingly independent to the anesthetic state; It is worth to note that the size of the correlation peak in the α band is much bigger in mPF-CC (and CC-S2) compared to other area pairs. In contrast, in imaging results the only difference is between the correlation time course of mPF-CC (which increased in time) and the other ones (which maintained stable values). Taken together, these findings seem to suggest a possible relationship between brain correlated activity measured from BOLD signal and coupled neural oscillation in the γ range.

# SUPPLEMENTAL METHODS

***Estimation of BOLD global synchronization.***

To compute the Kuramoto order parameter χ, we first extracted the BOLD signal of each ROI corresponding to each 10 minutes sliding window (each one corresponding to T = 200 sample points) for all rats and computed the Hilbert envelope of the bandpassed (0.04-0.07 Hz, Glerean at al. 2012) signal. The time-varying overall network synchronization within each sliding window was calculated as

$$\chi(t)=\frac{1}{N}\left(\sum_{j=1}^{N} e^{imag\theta_j(t)}\right) \quad , \ t = \{1, 2, \dots , \mathrm{T}\} \tag{1}$$

where $\theta_j(t)$ is the phase of the oscillation of each j-esim area at time *t,* being *N* the total number of areas.

***Detection of functional communities and modularity.***

In mathematical terms, being $C_i$ the community to which node *i* is assigned, Q is defined using the weighted adjacency matrix $W_{ij}$ , representing the strength of the edge between the nodes *i* and *j* (but set to 0 if no links exists between the two), as

$$Q=\frac{1}{2\mathrm{w}}\sum\sum\left(w_{ij}-\frac{w_i w_j}{2\mathrm{w}}\right)\delta(C_i,C_j) \tag{2}$$

where $\delta(C_i,C_j)$ is the Kronecker delta function, which takes 1 if nodes *i* and *j* are in the same community and 0 otherwise, the strengths $w_i=\sum w_{ij}$ , and the total strength $w=\sum w_i=\sum\sum w_{ij}$ .

***LFP data analysis.***

The difference between the BLCs of the two states (deep and light) was computed separately for each 1 Hz-width frequency band (*f*) for coupled and uncoupled area pairs *ij*, as

$$\Delta_{LD} r_{ij}(f)=[(r_{Light}(f)-r_{Deep}(f))]_{ij} \tag{3}$$

***Quantification of the frequency shift to higher frequencies.***

The relative correlation used to quantify the BLC frequency shift to higher frequencies was calculated as

$$rel\ r_{HL}(s)=\frac{r_{f_H}}{r_{f_L}}(s)\ ,\ s=\{1, 2, \ldots, S\}, \qquad (4)$$

where $r_{f_H}$ is the mean correlation of the higher component (e.g., 11-15 Hz), $r_{f_L}$ that of the lower one (e.g. 8-11 Hz) and *s* is a given sliding window. Each relative correlation coefficient we obtained was then converted to the corresponding Fisher's *z-score. s*
